# Reliabilities of mean and variability of ambulatory pain among community dwelling older adults

**DOI:** 10.1093/geroni/igab046.3221

**Published:** 2021-12-17

**Authors:** Jinshil Hyun, Jiyue Qin, Cuiling Wang, Mindy Katz, Jelena Pavlovic, Carol Derby, Richard Lipton

**Affiliations:** Albert Einstein College of Medicine, Bronx, New York, United States

## Abstract

Individual’s pain experiences vary substantially over time periods, and the variability in pain may be an important metric to predict health consequences. However, research on its reliability is lacking among older adults. We aimed to examine the reliabilities of both intra-individual mean (IIM) and intra-individual variability (IIV) of subjective pain reports assessed using ecological momentary assessments (EMA) among racially diverse, community dwelling older adults. Participants were from the Einstein Aging Study (N=311, age=70-91) and completed a 14-day EMA protocol which included self-reports of pain intensity 6 times a day. Pain IIV was quantified using intraindividual standard deviation (iSD). We followed Wang and Grimm(2012)’s approach to calculate the reliability of IIM and IIV. Over a 2-week period, we found excellent reliabilities for both pain IIM (.99) and pain IIV (.91), showing that these measures are reliable and can be used to link with various health outcomes among community dwelling older adults. We also estimated the average number of assessments that produce acceptable levels of reliability. The average of 2 assessments for pain IIM and 23 assessments for pain IIV produced values that exceeded reliability score of .80, suggesting that a briefer study design may be used to reduce participants’ burden with reliable pain metrics. Future studies need to examine whether pain IIV is associated with cognitive, emotional, and physical health among older adults and whether intervention studies that target to reduce pain IIV improve health consequences.

